# Direct Interaction Between Roe Deer and Mountain Hare, a Case of Interference Encounter Competition?

**DOI:** 10.1002/ece3.73771

**Published:** 2026-06-01

**Authors:** Simen Pedersen, Synnøve Hervik, Hans Christian Pedersen

**Affiliations:** ^1^ Department of Forestry and Wildlife Management, Faculty of Applied Ecology, Agricultural Sciences and Biotechnology, Campus Evenstad University of Inland Norway Koppang Norway; ^2^ Faculty of Environmental Sciences and Natural Resource Management Norwegian University of Life Sciences Ås Norway; ^3^ Department of Terrestrial Biodiversity Norwegian Institute for Nature Research Trondheim Norway

**Keywords:** camera trapping, *Capleolus capreolus*, competition, *Lepus timidus*

## Abstract

Roe deer and mountain hares are known to select the same food sources and engage in indirect exploitative competition. Here we report on a case of direct interaction between a roe deer doe and a mountain hare recorded by a camera trap in Tydal Municipality, Norway, a possible case of direct interference competition. The roe deer doe is at least following and displacing the mountain hare for 2 min and 29 s, including a 19 s active chase phase. The two animals can be seen circling in and out of frame in front of the camera. This direct interaction is based on the limited information gained through the picture series. It could be a case of curiosity; however, we speculate that this is a case of interference encounter competition between these two herbivore species. From the literature, roe deer and mountain hares are known to compete indirectly for forage; moreover, roe deer are known to behave aggressively towards other competing deer species. Our traditional textbook understanding of ecology is based on the common and the easily observable, not the rare and difficult to observe. This observation challenges our understanding of the interactions between roe deer and hares, as roe deer may compete directly with mountain hares over food resources.

## Graphical abstract

Here, we report on a case of direct interaction between a roe deer doe and a mountain hare recorded by a camera trap in Tydal Municipality, Norway, suggestive of direct interference competition. The roe deer doe is following and displacing the mountain hare for at least 2 min and 29 s, including a 19 s active phase. This observation challenges our understanding of the interactions between roe deer and hares, as roe deer may compete directly with mountain hares over food resources.

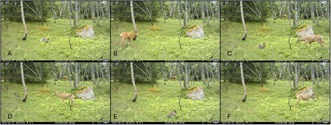

## 1 Introduction

Interspecific interactions among mammalian herbivores range from (1) direct or interference competition (Schoener 1983), (2) indirect or exploitative competition (Schoener 1983), to (3) forage facilitation (Arsenault and Owen‐Smith 2002) and (4) multispecies herds with shared vigilance for predators (Stears et al. 2020). According to niche theory, food competition occurs only if there is high overlap in use of limited forage resources. Herbivores are not expected to compete if they differ in body size (Demment and van Soest 1985) or if they differ in digestive systems (Duncan et al. 1990). However, due to an interaction between different digestive systems (foregut fermenters versus hindgut fermenters) and different body mass (large versus small), large ruminants such as cervids may compete with small hindgut fermenters such as hares (Belovsky 1984; Hofmann 1989; Johannessen and Samset 1994; Hulbert and Andersen 2001; Öhmark et al. 2015; Sangiuliano et al. 2016; Pedersen and Pedersen 2021). Roe deer ( *Capreolus capreolus* ) and mountain hares ( *Lepus timidus* ) have previously been shown to compete indirectly over food resources during winter, expressing exploitative competition (Hulbert and Andersen 2001). Moreover, mountain hares are competitively inferior to roe deer as they have a lower maximum browsing height, and Hulbert and Andersen (2001) suggest that this may cause roe deer to limit mountain hare populations, especially in island systems. Furthermore, roe deer and European hares ( *L. europaeus* ) are known to compete indirectly for forage (Sangiuliano et al. 2016), and moreover European hares avoid areas of high roe deer abundance (Viviano et al. 2021). However, Schwegmann et al. (2025) found support for the intermediate disturbance hypothesis of roe deer densities on European hares, suggesting a negative effect of roe deer, but only at higher densities. Reiterating the continuum of the four types of interactions among herbivores, roe deer and mountain hares would, based on the current knowledge, best be described as a case of indirect or exploitative competition (Schoener 1983). Here we report on a case of a roe deer doe following and displacing a mountain hare, a possible case of interference encounter competition between the two species.

## 2 Methods

The study area is situated in Tydal Municipality, Trøndelag County, Norway (63.0° N, 11.7° E) between 600 and 900 m.a.s.l. and is dominated by downy birch (*Betula pubeschens*), interspersed with bogs, fields and Scots pine ( *Pinus sylvestris* ) and Norway spruce ( *Picea abies* ) forest. Other tree species like rowan ( *Sorbus aucuparia* ) and willow (*Salix* spp.) are also present at lower abundance. The shrub layer consists of graminoids such as *Poa* spp., *Carex* spp., *Equisetum* spp., *Juncus* spp., herbs such as *Melampyrum* spp., *Epilobium* spp., ferns such as *Gymnocarpium* spp., and dwarf shrubs such as bilberry ( *Vaccinium myrtillus* ) and crowberry ( *Empetrum nigrum* ). The main species of the vertebrate herbivore community are mountain hares, willow ptarmigan ( *Lagopus lagopus* ), capercaillie ( *Tetrao urogallus* ), black grouse ( *Lyrurus tetrix* ), roe deer, semidomestic reindeer ( *Rangifer tarandus* ) and moose ( *Alces alces* ) as well as voles (*Microtus* spp., *Myodes glareolus*). Main hare predators include red fox ( *Vulpes vulpes* ), lynx ( *Lynx lynx* ), Eurasian goshawk (*Astur gentilis*), golden eagle ( *Aquila chrysaetos* ), Eurasian eagle owl ( *Bubo bubo* ). The mean daily temperature in Tydal ranges from −28.5°C to 22.2°C (2004–2025 data from www.senorge.no). Annual precipitation is around 900 mm (Rekdal 2013).

As part of a project on behavioural responses to coat colour mismatch (HareKlima), we deployed 12 camera traps (Reconyx Hyperfire 4K Professional WhiteFlash) to monitor coat phenology of individually GPS marked mountain hares. Camera traps were placed in locations where GPS tagged hares frequently passed, with the aim of documenting individual moult phenology. Camera traps were operational between February 25th 2025 and December 27th 2025, for a total of 306 days. No bait, lure or any other attractant was used. Cameras were programmed to rapidly take continuous pictures if triggered by movement. A total of 350 hare and 137 roe deer observations were recorded across all 12 camera traps, with 10 cameras capturing both species, while two cameras captured mountain hares only. There were no other records of hares and roe deer together on the same pictures. The one camera we report from here was located at 62.95° N, 11.77° E.

## 3 Results

**FIGURE 1:**
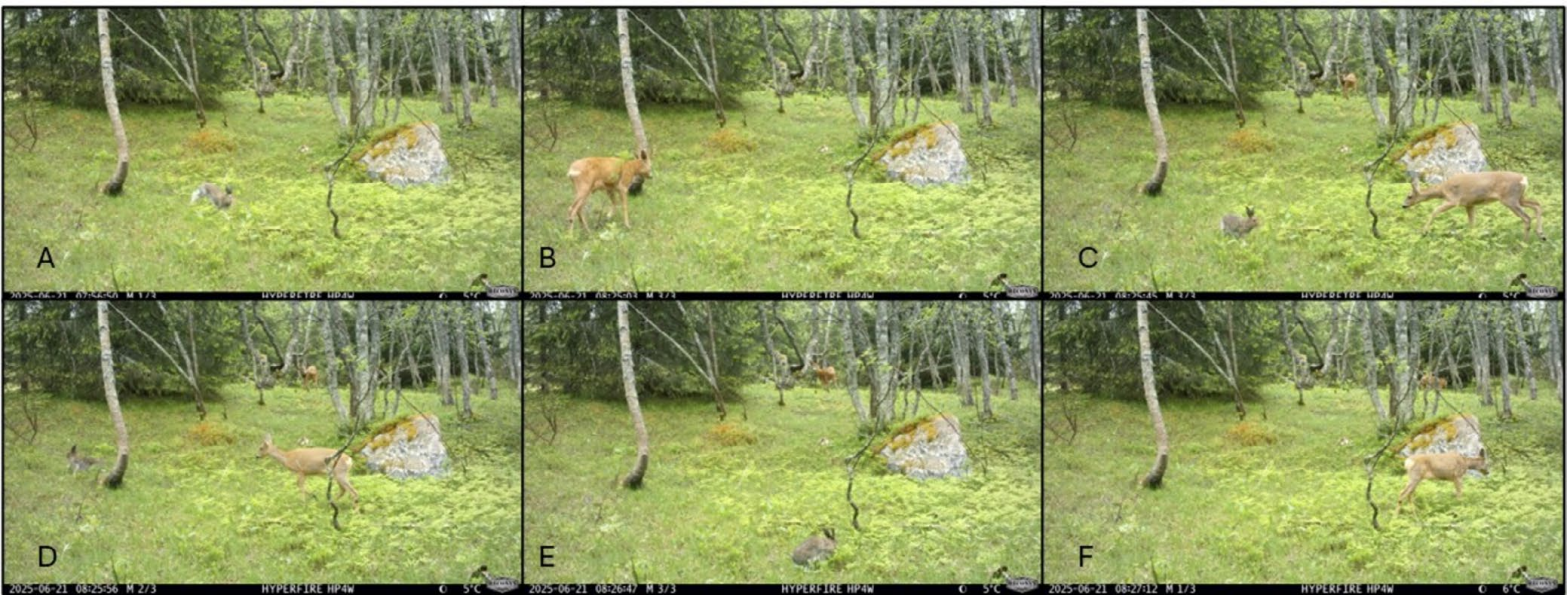
Interaction between a roe deer ( *Capreolus capreolus* ) doe and a mountain hare ( *Lepus timidus* ) on June 21st 2025, in Tydal Municipality, Trøndelag County, Norway. (A) 07:56:50: The mountain hare passed in front of the cameratrap. (B) 08:25:01: Roe deer buck passing in front of the cameratrap. (C) 08:25:43: Start of interaction between the roe deer doe and the mountain hare. (D) 08:25:56: The roe deer displaces the mountain hare once again and both disappear out of frame. (E) 08:26:46: The hare re‐enters the frame and appears to be foraging. (F) 08:27:14: The roe deer follows the path of the hare and leaves the frame. Total interaction lasted at least 2 min 29 s, while the active chase lasted at least 19 s. See Supporting Information for a video of all still pictures.

On June 21st 2025 at 07:56:50, an unmarked mountain hare passed in front of the camera trap, without stopping (Figure 1A, Video S1). From 08:25:01 to 08:25:10 there is a sequence of nine pictures of a roe deer buck walking in front and away from the camera trap (Figure 1B). At 08:25:43 the roe deer buck can be seen in the background, while a mountain hare and a roe deer doe are in the foreground (Figure 1C). We judge the roe deer doe to be a yearling based on body shape, and general appearance. The roe deer doe approaches the mountain hare, until she is approximately 1.5–2.0 m from the hare (estimated from the picture, and based on average dimensions of roe deer). The hare runs off towards the camera trap and to the right and disappears out of frame. The roe deer appears to follow the hare, and the hare re‐enters the frame, and stops (seemingly to forage on an unknown forage plant). The roe deer doe continues to approach the hare, the hare runs off again, this time at a longer distance from the roe deer (estimated distance 2–3 m) (Figure 1D). Both the hare and the roe deer leave the picture frame at 08:26:01. At 08:26:46 the hare re‐enters the picture frame from the lower left corner and appears to be foraging (head towards the ground) before leaving the picture frame to the right at 08:26:53 (Figure 1E). Seven seconds later at 08:27:00 the roe deer doe approaches from the left, nibbles a rowan leaf and continues to the right, following the path of the hare (Figure 1E). The roe deer doe leaves the frame at 08:27:14. From 08:27:52 to 08:27:54 the hare can be seen moving from the right to the left, this time without being followed by the roe deer (Figure 1F). No further activity was recorded on June 21st 2025. Based on what we document through the frame of the camera trap, the whole interaction between the roe deer and the hare lasted at least for 2 min and 29 s. The ‘active phase’, where the roe deer is a few meters from the hare and the hare can be seen moving away from the roe deer lasted at least 19 s. What is interesting to note is that most likely the hare, and definitely the roe deer, were feeding within the patch.

## 4 Discussion

Through camera trapping we document a rare interaction between a roe deer doe and a mountain hare, where the roe deer follows and displaces the hare repeatedly, over at least 2 min and 29 s. We acknowledge the limitations of this observation. Despite the continuous picture series, still pictures give limited information compared to, for example, direct observation or video and sound that would better capture any aggressive or defensive behaviours from the interacting animals. This documented direct interspecific interaction may be interpreted several ways, at one end of the spectrum it could be a case of curiosity or playfulness from the side of the roe deer (Byrne 2013) followed by a startle response in the hare, at the other extreme this may be a predation attempt (Pietz and Granfors 2000; Vazquez et al. 2023). We however suggest the most plausible is in between these two extremes, namely a case of interference encounter competition (Schoener 1983). We base this on roe deer and mountain hares being described in the literature as competing indirectly for food resources (Hulbert and Andersen 2001). Furthermore, roe deer show interspecific aggression towards the larger fallow deer ( *Dama dama* ) under food competition (Ferretti 2011).

Food competition occurs if there are limited forage resources. Based on this one would expect winter season with snow covering the field layer vegetation to be the time of high competition between roe deer and mountain hare (Hulbert and Andersen 2001). However, the observed interaction occurred after initiation of the growing season in spring. This contradicts the interaction being a case of interference competition, since food plants should be abundant. However, many species have an established ‘enemy view’ and will displace any competing species when encountered, even if resources are abundant (Schoener 1983).

Our traditional textbook understanding of ecology is based on the common and the easily observable, not the rare and difficult to observe. As pointed out in their study on roe deer and mountain hare food competition, Hulbert and Andersen (2001) state ‘…there was no evidence from personal observation or from the literature of either species interacting with the other in an antagonistic manner, e.g. chasing the other’. Direct encounters of roe deer and mountain hares are most likely infrequent and thus seldom observed, but that does not mean they are unimportant for structuring their foraging behaviour and space use (Viviano et al. 2021). As a comparison, it is well known that the mountain hare is a prey species, and predation is an important interaction shaping its life history, morphology, behaviour and space use. Despite this, we did not record a single predation event at any of the camera traps, but that does not mean predation is not an important interaction in this system. We speculate that established dominance hierarchies of competing species likely do not cause frequent aggressive behaviours, since individuals of both species are aware of their own dominance status. Thus, such direct interactions will infrequently be observed, as the dominant species displaces the subordinate without aggression. Indeed, Ferretti (2011) found roe deer and fallow deer to directly interact, but showed aggression in a limited number of interactions.

Studies of seasonal diet overlap between the two interacting species would provide insights to the scope for food competition between the two, and how this may change with seasons. There are several studies of winter food choice of both species in Scandinavia, but studies of summer diet are limited (see Cederlund et al. 1980; Johannessen and Samset 1994). Unfortunately, no studies to our knowledge have investigated degree of diet overlap in allopatric and sympatric populations; this would be a key area of future research.

This case is yet another example of how the development of new technologies such as GPS collars and camera traps allows us to monitor animals for an extended amount of time, and with a detail level previously not possible. Thus giving us insights that previously were hidden (e.g., Lai et al. 2022; Spitzer et al. 2023; Pedersen et al. 2026). This observation challenges our understanding of the interactions between roe deer and hares, as roe deer may compete directly with mountain hares over food resources.

## Author Contributions

**Simen Pedersen:** conceptualization (lead), funding acquisition (lead), investigation (equal), writing – original draft (lead), writing – review and editing (equal). **Synnøve Hervik:** data curation (lead), investigation (equal), writing – review and editing (equal). **Hans Christian Pedersen:** investigation (equal), writing – review and editing (equal).

## Funding

This work was supported by the Trygve Gotaas Fond, the Norwegian Environmental Agency (grant no. 2023/1082, 2024/805) and the Norwegian Agricultural Agency.

## Conflicts of Interest

The authors declare no conflicts of interest.

## Supporting information


**Video S1:** Video sequence of all still photos capturing the interaction between a roe deer ( *Capreolus capreolus* ) doe and a mountain hare ( *Lepus timidus* ) on June 21st 2025, in Tydal Municipality, Trøndelag County, Norway. Total interaction lasted at least 2 min 29 s, while the active chase lasted at least 19 s.

## Data Availability

No data except enclosed pictures and animation in Supporting Information was used in the creation of this paper.

## References

[ece373771-bib-0001] Arsenault, R. , and N. Owen‐Smith . 2002. “Facilitation Versus Competition in Grazing Herbivore Assemblages.” Oikos 97: 313–318.

[ece373771-bib-0002] Belovsky, G. E. 1984. “Moose and Snowshoe Hare Competition and a Mechanistic Explanation From Foraging Theory.” Oecologia 61: 150–159. 28309404 10.1007/BF00396753

[ece373771-bib-0003] Byrne, R. W. 2013. “Animal Curiosity.” Current Biology 23: R469–R470. 23743408 10.1016/j.cub.2013.02.058

[ece373771-bib-0004] Cederlund, G. , H. Ljungqvist , G. Markgren , and F. Stalfelt . 1980. “Foods of Moose and Roe‐Deer at Grimsö in Central Sweden. Results of Rumen Content Analyses.” Viltrevy 11: 169–244.

[ece373771-bib-0005] Demment, M. W. , and P. J. van Soest . 1985. “A Nutritional Explanation for Body‐Size Patterns of Ruminant and Nonruminant Herbivores.” American Naturalist 125: 641–672.

[ece373771-bib-0006] Duncan, P. , T. J. Foose , I. J. Gordon , C. G. Gakahu , and M. Lloyd . 1990. “Comparative Nutrient Extraction From Forages by Grazing Bovids and Equids—A Test of the Nutritional Model of Equid Bovid Competition and Coexistence.” Oecologia 84: 411–418. 28313034 10.1007/BF00329768

[ece373771-bib-0007] Ferretti, F. 2011. “Interspecific Aggression Between Fallow and Roe Deer.” Ethology Ecology & Evolution 23: 179–186.

[ece373771-bib-0008] Hofmann, R. R. 1989. “Evolutionary Steps of Ecophysiological Adaptation and Diversification of Ruminants—A Comparative View of Their Digestive‐System.” Oecologia 78: 443–457. 28312172 10.1007/BF00378733

[ece373771-bib-0009] Hulbert, I. A. R. , and R. Andersen . 2001. “Food Competition Between a Large Ruminant and a Small Hindgut Fermentor: The Case of the Roe Deer and Mountain Hare.” Oecologia 128: 499–508. 28547395 10.1007/s004420100683

[ece373771-bib-0010] Johannessen, V. , and E. Samset . 1994. “Summer Diet of the Mountain Hare (*Lepus timidus* L) in a Low‐Alpine Area in Southern Norway.” Canadian Journal of Zoology‐Revue Canadienne De Zoologie 72: 652–657.

[ece373771-bib-0011] Lai, S. , É. Desjardins , J. Caron‐Carrier , et al. 2022. “Unsuspected Mobility of Arctic Hares Revealed by Longest Journey Ever Recorded in a Lagomorph.” Ecology 103: e3620. 34939184 10.1002/ecy.3620

[ece373771-bib-0012] Öhmark, S. M. , G. R. Iason , and R. T. Palo . 2015. “Spatially Segregated Foraging Patterns of Moose ( *Alces alces* ) and Mountain Hare ( *Lepus timidus* ) in a Subarctic Landscape: Different Tables in the Same Restaurant?” Canadian Journal of Zoology 93: 391–396.

[ece373771-bib-0013] Pedersen, S. , and H. C. Pedersen . 2021. “Exploitative Competition Between Mountain Hare and Moose‐Qualitative Effects on Hare Winter Forage?” Animals 11: 2638. 34573604 10.3390/ani11092638 PMC8469073

[ece373771-bib-0014] Pedersen, S. , H. C. Pedersen , S. Kalleberg , and M. Mayer . 2026. “Long‐Distance Dispersal in Mountain Hare Revealed by GPS Collaring.” Ecology 107: e70414. 42135609 10.1002/ecy.70414

[ece373771-bib-0015] Pietz, P. J. , and D. A. Granfors . 2000. “White‐Tailed Deer ( *Odocoileus virginianus* ) Predation on Grassland Songbird Nestlings.” American Midland Naturalist 144: 419–422.

[ece373771-bib-0016] Rekdal, Y. 2013. Vegetasjon og Beite i tre Utmarksområde i Tydal Kommune—Vessingsjøen, Kranklia og Hyllingen. Skog og Landskap.

[ece373771-bib-0017] Sangiuliano, A. , S. Lovari , and F. Ferretti . 2016. “Dietary Partitioning Between European Roe Deer and European Brown Hare.” European Journal of Wildlife Research 62: 527–535.

[ece373771-bib-0018] Schoener, T. W. 1983. “Field Experiments on Interspecific Competition.” American Naturalist 122: 240–285.

[ece373771-bib-0019] Schwegmann, S. , J. M. C. Pereira , M. Basile , et al. 2025. “Testing the Intermediate‐Disturbance Hypothesis—Managed Roe Deer Populations Are Not Disrupting Forest Faunal Communities.” Basic and Applied Ecology 89: 1–12.

[ece373771-bib-0020] Spitzer, R. , C. Åström , A. Felton , et al. 2023. “Coprophagy in Moose: A First Observation.” Ecology and Evolution 13: e9757. 36699571 10.1002/ece3.9757 PMC9852938

[ece373771-bib-0021] Stears, K. , M. H. Schmitt , C. C. Wilmers , and A. M. Shrader . 2020. “Mixed‐Species Herding Levels the Landscape of Fear.” Proceedings of the Royal Society B‐Biological Sciences 287: 20192555. 10.1098/rspb.2019.2555 PMC7126070 32126952

[ece373771-bib-0022] Vazquez, M. S. , D. Gonzalez , and G. C. Amico . 2023. “Herbivores but Not Vegans: Deer as Nest Predators.” Austral Ecology 48: 1460–1465.

[ece373771-bib-0023] Viviano, A. , E. Mori , N. Fattorini , et al. 2021. “Spatiotemporal Overlap Between the European Brown Hare and Its Potential Predators and Competitors.” Animals 11: 562. 33669965 10.3390/ani11020562 PMC7924828

